# PDS5A and PDS5B in Cohesin Function and Human Disease

**DOI:** 10.3390/ijms22115868

**Published:** 2021-05-30

**Authors:** Nenggang Zhang, Luiza E. Coutinho, Debananda Pati

**Affiliations:** Department of Pediatrics, Texas Children’s Cancer Center, Baylor College of Medicine, 1102 Bates Street, Houston, TX 77030, USA; nzhang@bcm.edu (N.Z.); luizaecoutinho@gmail.com (L.E.C.)

**Keywords:** PDS5A, PDS5B, cohesin, cancer, cohesinopathy

## Abstract

Precocious dissociation of sisters 5 (PDS5) is an associate protein of cohesin that is conserved from yeast to humans. It acts as a regulator of the cohesin complex and plays important roles in various cellular processes, such as sister chromatid cohesion, DNA damage repair, gene transcription, and DNA replication. Vertebrates have two paralogs of PDS5, PDS5A and PDS5B, which have redundant and unique roles in regulating cohesin functions. Herein, we discuss the molecular characteristics and functions of PDS5, as well as the effects of its mutations in the development of diseases and their relevance for novel therapeutic strategies.

## 1. Introduction

Cohesin plays essential roles in a variety of cellular processes, including sister chromatid cohesion, repair of damaged DNA, gene transcription, DNA replication, centrosome biogenesis, and maintenance of genome integrity [1,2,3,4,5,6,7,8,9,10]. It is a conserved multiprotein complex with three core subunits—two structural maintenance of chromosomes (SMC) proteins (SMC1 and SMC3) and an α-kleisin (RAD21/MCD1/SCC1)—forming an annular trimer [8,11,12,13]. Both SMC1 and SMC3 fold into intramolecular antiparallel coiled coils with an ATPase-containing head domain on one end and a hinge domain on the other end. The ATPase-containing head is formed with the N- and C-termini of each SMC molecule, and the hinge domain is from its middle region [14]. SMC1 and SMC3 join together through their hinge domains, creating a V-shaped heterodimer with the ATPase-containing heads at the distal end of the two coiled-coils arms [14,15,16]. The ATPase-containing head domains of SMC1 and SMC3 together form a composite ABC-like ATPase that consists of Walker A and Walker B motifs of one head and the Signature motif of the opposite head. Two molecules of ATP can bind to the composite ABC-like ATPase [14,17]. The C-terminus of RAD21 binds to the ATPase-containing head of SMC1, and the N-terminus of RAD21 binds to the coiled coil next to the ATPase-containing head of SMC3, resulting in the formation of a tripartite ring [14,18]. Besides those three core subunits, cohesin-associated proteins, such as SCC3, PDS5, and WAPL, bind to the cohesin core complex via RAD21 [19,20,21,22,23].

Cohesin functions in both mitotic and meiotic cells, but some subunits of the cohesin in these two types of cells are different. In mammal, SMC1A, SMC3, RAD21, and STAG1/STAG2 are cohesin subunits found in mitotic cells, which form two types of cohesin complex because of two STAG proteins (STAG1/STAG2). In meiotic cells, SMC1B, Rad21/REC8/RAD21L, and STAG3 are the components of cohesin complex, which form three different cohesins because there are three different α-kleisins [24].

In dividing cells, cohesin complexes are loaded to chromatin at telophase by a cohesin-loading complex that consists of NIPBL/MAU2 heterodimer in humans and SCC2/SCC4 in yeast. NIPBL and SCC2, as well as MAU2 and SCC4 are orthologous proteins. Cohesin dynamically associates with chromatin until the S phase, when DNA is replicated. The sister chromatids are held together by the cohesin complexes, and sister chromatid cohesion is generated after SMC3 is acetylated by cohesin acetyltransferases (CoATs) (Eco1/Ctf7 in yeast or ESCO1/2 in human) [25,26,27,28]. PDS5 plays important roles in the generation, maintenance, and resolution of sister chromatid cohesion. In vertebrates, PDS5, sororin, and WAPL form a cohesin-regulatory complex, in which sororin and WAPL compete to bind to a specific site on PDS5 to regulate the association of cohesin on chromatin [23,29,30,31,32,33,34]. The cohesin-regulatory complex regulates the association of cohesin with chromosomes either positively or negatively depending on which protein binds to the site on PDS5. PDS5-sororin complex maintains sister chromatid cohesion from the S phase to G2, whereas PDS5-WAPL complex dislodges cohesin from chromatin. After WAPL displaces sororin that is phosphorylated by kinases, such as Cdk1/Cyclin B and Plk1 in the prophase, a PDS5-WAPL complex is formed, which then plays a role in releasing cohesins from chromosome arms. The cohesins on centromeres and residual arm cohesins are removed by separase through cleaving RAD21 on the transition of metaphase and anaphase, resulting in the sister chromatids being segregated into daughter cells [35,36,37,38,39,40].

There are two paralogs of PDS5, PDS5A and PDS5B, in vertebrates. Limited studies indicate that they have common and specific roles in cell growth and development. Although PDS5 plays important roles in regulating functions of the cohesin complex, it has not attracted much attention in the cohesin field. Herein, we review the protein characteristics of PDS5 and its role in regulating cohesin functions in mitotic cells, as well as the implication in human disease.

## 2. Identification and Characteristics of PDS5

Cohesin is a versatile protein complex that is involved in various cellular processes by associating different regulatory partners. In efforts to identify more cohesin-interacting proteins that are required to elucidate the mechanisms on sister chromatid cohesion and condensation, *SPO76* gene cloned from *Sordaria macrospora* [41] and *bimD* from *Aspergillus nidulans* [42] drew the attention of researchers in the cohesin biology field [43,44] for their roles in mitotic and meiotic chromosome segregation [42,45]. Genetic and functional analyses indicated that PDS5 is a cohesin-associated protein and is essential for the cohesin to function. It is homologous to Spo76p and BIMD and conserved from fungi to human [43,44,45].

**Figure 1:**
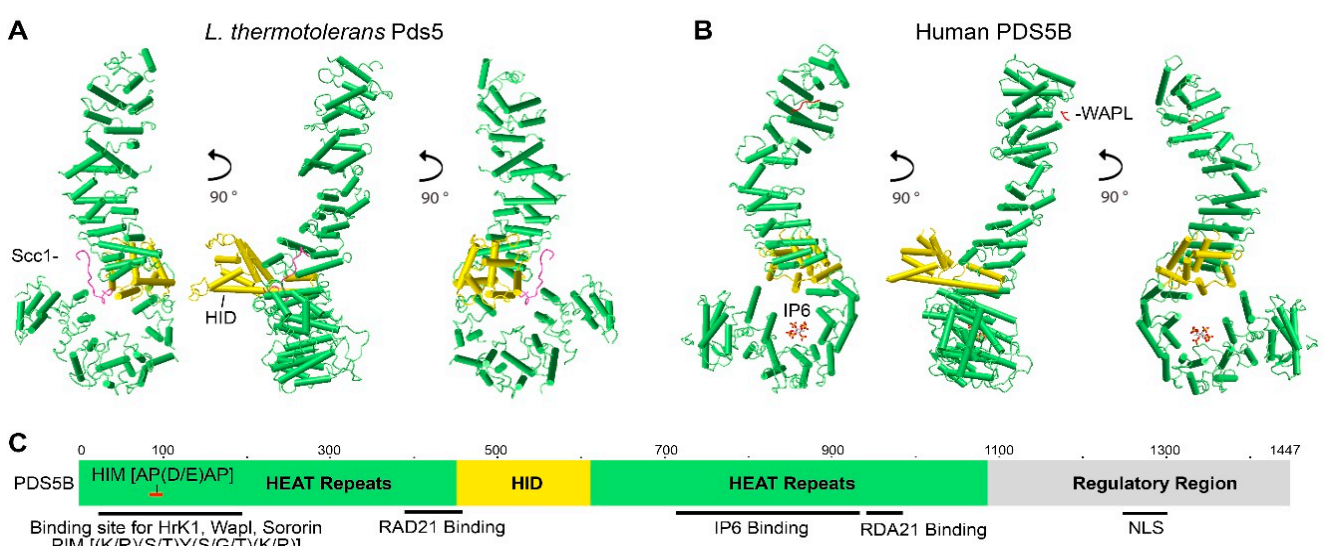
Structure and characteristics of the PDS5 protein. Crystal structure images of (**A**) *L.* *thermotolerans* Pds5 (PDB ID: 5F0O) and (**B**) human PDS5B (PDB ID: 5HDT) were generated by using NCBI’s web-based 3D structure viewer iCn3D. PDS5 is a hook-like molecule consisting of HEAT repeats (green sticks) with helical insert domain (HID, golden sticks) extruding on one side of the hook. Binding of Scc1/Rad21 (pink line) to *L.* *thermotolerans* Pds5 is shown in (**A**). Binding of WAPL (red line) to the C-terminus of human PDS5B and IP6 to the bottom of PDS5B hook-like structure are shown in (**B**). (**C**) Schematic drawing shows PDS5 molecular features. The relative site is based on human PDS5B. Hrk1 interacting motif (HIM) on PDS5 N-terminus interacts with the PDS5 interacting motif (PIM) on HrK1, WAPL, and sororin. RAD21 and IP6 interact with PDS5 in the middle region. Nuclear localization signal (NLS) is on the C-termini of PDS5A and PDS5B (Refer to Figure 2).

PDS5 proteins contain more than 20 HEAT repeats (each is composed of two alpha helices linked by a short loop), forming two clusters separated by a helical insert domain (HID) (Figure 1) [44,46,47,48]. Multiple HEAT repeats form extended superhelical structures and can function as scaffolds to facilitate the assembly of other molecular components. Similar to PDS5, cohesin subunit SCC3 (STAG1, STAG2) and cohesin-associated proteins, such as SCC2, WAPL, and separase, also consist of HEAT repeats. [20,21,49,50,51,52,53,54,55].

Full-length PDS5 proteins from *Lachancea thermotolerans*, *Saccharomyces cerevisiae*, and humans are comprised of 1292aa, 1277aa, and 1347aa, respectively. Crystal structures for truncated *L.* *thermotolerans* PDS5 (45-1221aa), *S.* *cerevisiae* Pds5 (1-701aa), and human PDS5B (21-1121aa) have been resolved (Figure 1A,B) [46,56,57]. Although yeast and human PDS5 have only approximately 20% identity at the amino acid residue level, they are conserved at the structural level. Both yeast Pds5 and human PDS5B are composed of alpha helical coils and look like a hook, with the stem or spine consisting of the HEAT repeats of the N-terminus and the hook consisting of the HEAT repeats of the middle and C-termini (Figure 1A,B) [46,56]. The hook tip bends back and contacts the middle of the spine, forming a ring-like space approximately 10Å in diameter [56]. Most of the HEAT repeats are on the spine [56,57]. The size of the hook structure is approximately 150Å [56].

The PDS5 protein has several interesting features at the sequence level. The N-terminus of PDS5 has a conserved serine/threonine-protein kinase Haspin/Hrk1-interacting motif (HIM) (A-P-D/E-A-P) that interacts with a PDS5-interacting motif (PIM) (K/P-S/T-Y-S/T/G-K/R) found on WAPL and Hrk1 from yeast to human, as well as yeast Eso1 of the *Schizosaccharomyces* group and sororin in vertebrates (Figure 1C) [30,46]. Human PDS5B binds to WAPL at the region of 1-33aa and to sororin at the region of 131–171aa. The PIM on both WAPL and sororin contains a YSR motif (with the consensus of [K/R] [S/T]YSR) that is conserved in vertebrates [46]. It has been reported that the region of the YSR motif on sororin interacts with cohesin complex, but to which cohesin-subunit or associated protein remains to be identified [33,34,58]. Mutations of the YSR motif in WAPL or sororin abolished their binding to purified PDS5B, suggesting that the YSR motif is crucial for WAPL and sororin to interact with PDS5 directly [46]. Interestingly, the FGF motif on sororin and two of the three FGF motifs on WAPL located downstream of the YSR motif are also important to bind to PDS5 [33,34,58].

Structural analysis of human PDS5B reveals that inositol hexakisphosphate (IP6) binds to the bottom of the PDS5 hook (Figure 1B,C) [46]. The basic amino acid residues with which the IP6 interacts are positively charged and conserved from yeast to humans. Mutations of those amino acid residues cause PDS5 deficiency in cohesin binding [46], implying that IP6 may contribute to maintaining the proper structure of PDS5 in order for it to interact with cohesin. IP6 and other higher inositol polyphosphates, such as IP5 and IP4, are abundant lipid-derived metabolites in eukaryotic cells and can function as structural cofactors for enzymes, receptors, and protein complexes [59,60,61]. Although IP6 has not been reported in the crystal structure study of yeast Pds5 [56], the abundance of IP6 in eukaryotic cells and the conservation of the PDS5 structure suggest that IP6 might also bind to PDS5 from other organisms and participate in protein-protein interactions.

Yeast Pds5 interacts with the N-terminus of Scc1/Rad21 (101-122aa in human, 120-141aa in yeast *L. thermotolerans*) at the opening of the hook (Figure 1A) [46,56,57]. This Pds5-interacting region on Scc1 is located just downstream of the domain where Scc1 binds to SMC3 [18]. Scc1 binds to Pds5 and is wedged between the spine and the hook, causing the opening of the hook [56].

Vertebrates have two paralogs of the PDS5 protein, PDS5A and PDS5B. In humans, PDS5A is located on the small arm of chromosome 4, at position 14 (4p14), and has 36 exons. PDS5B is on the long arm of chromosome 13, at position 13.1 (13q13.1), and has 38 exons. PDS5A and PDS5B consist of 1337aa and 1447aa, respectively, with an overall sequence identity of 70%. The N-termini of PDS5A and PDS5B (1-1200aa) have approximately 72% sequence identity, whereas the C-termini (from 1201aa to the end) are predominantly different (sharing only less than 28% identity). Therefore, it is possible that the N-termini determine their common function, whereas the C-termini differentiate their roles in the cell cycle.

**Figure 2:**
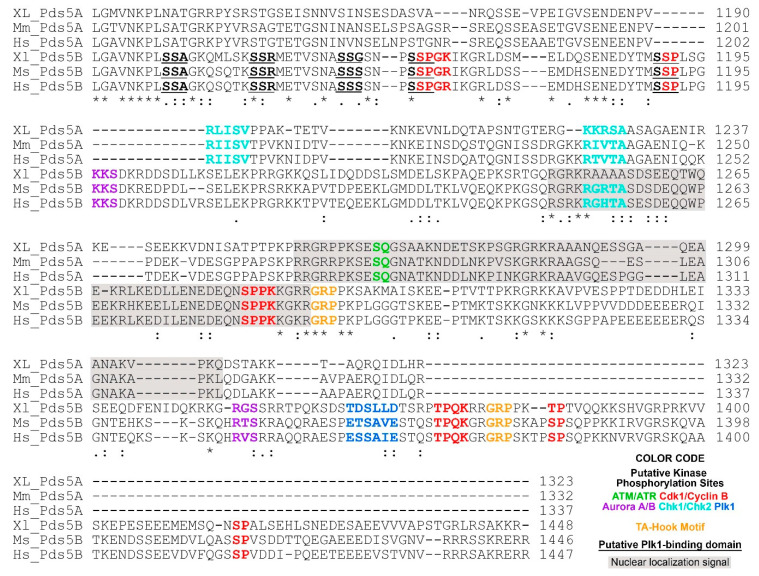
C-terminal alignment of PDS5A and PDS5B from frog (*Xenopus*), mouse and human. The following putative regulatory features are highlighted in color: ATM/ATR phosphorylation motif (S/T)-Q [62]; Aurora A/B phosphorylation motif (R/K)_1-3_-X-(S/T)-(^P) where X is any amino acid, and ^P is not proline [63,64,65]; Cdk1/Cyclin B phosphorylation motif minimum consensus sequence (S/T)-P, full consensus sequence (S/T)-P-X-(K/R) [66]; Chk1/Chk2 phosphorylation motif (Φ/B)-(X/B)-(R/K)-X-X-(S/T)-Φ where B is a basic amino acid and Φ is a hydrophobic amino acid [67]; Plk1 phosphorylation motif (D/E)-X-(S/T)-Φ-X-(D/E). Plk1 binding domain (PBD) S-(pS/pT)-(P/X) where pS is a phosphorylated serine, pT is a phosphorylated threonine [68]. PBD is underlined. AT-hook motif GRP is highlighted in orange [69]. Nuclear localization signals are predicated using cNLS Mapper [70] and shaded in gray. The alignment was performed using Clustal O (1.2.4) [71]. Invariant, conserved and semi-conserved residues are indicated by an asterisk (*), colon (:) and period (.), respectively. The abbreviations are XL, *Xenopus laevis*; Mm, *Mus musculus*; Hs, *Homo sapiens*. Protein sequences are from NCBI and the access reference numbers are NP_001090063.1 (XL-PDS5A); NP_001089658.1 (XL-PDS5B), NP_001074790.1 (Mm-PDS5A), NP_780519.3 (Mm-PDS5B), NP_001093869.1 (Hs-PDS5A), NP_055847.1 (Hs-PDS5B).

Several interesting features were revealed after close examination of the C-termini of PDS5A and PDS5B from frog (*Xenopus*), mouse, and human sequences (Figure 2). (1) Nuclear localization signals are located on the C-terminus and conserved in PDS5A and PDS5B from different species. (2) PDS5A has two putative Chk1/Chk2 phosphorylation sites on its C-terminus, which are conserved in *Xenopus*, mice, and humans. Although one of the Chk1/Chk2 putative phosphorylation sites is also found on PDS5B in mice and humans, it is mutated in *Xenopus* PDS5B (Figure 2). (3) PDS5A has one putative ATM/ATR phosphorylation site, which does not present on PDS5B. (4) PDS5B has two putative Aurora A/B phosphorylation sites, six Cdk1/cyclin B putative phosphorylation sites, and one putative Plk1 phosphorylation site, as well as five Plk1 putative binding sites [S-(pS/pT)-P] (Figure 2). Phosphorylation of many of the sites have been detected in cells treated with UV or IR [72].

Two AT-hook motifs are present in PDS5B (Figure 2) [73,74,75,76]. The core amino acid residues of the AT-hook motif is GRP [69]. Proteins containing AT-hook motifs can bind to the minor groove of adenine-thymine (AT)-rich DNA. The optimal sequences for AT-hook proteins to bind are (TATT)*_n_* or (AATA)*_n_* repeat with AA(T/A)T at its center [77]. PDS5B can bind to DNA, and mutations of AT hook motifs abolish the binding [75]. However, the results from a study to identify which part of PDS5B contributes to the binding to DNA indicate that the DNA-binding property of PDS5B is not from the AT-hook-containing C-terminus [76]. It is possible that the reason PDS5B AT-hook motifs fail to bind to DNA is because the DNA used in this study lacks AT repeats. In summary, PDS5A and PDS5B are highly conserved proteins except for their C-termini, suggesting that the common functions of PDS5A and PDS5B may be directed through their N-termini, whereas their C-termini play regulatory roles corresponding to their specific functions.

## 3. PDS5 Functions

### 3.1. Role in Sister Chromatid Cohesion and Separation

PDS5 regulates cohesin functions by promoting the establishment, maintenance, and resolution of sister chromatid cohesion. It achieves these roles, sometimes contradictory in nature (promotion and resolution of cohesion), by partnering with other cohesin regulatory proteins including Eco1/Esco1/Esco2, sororin and WAPL. PDS5 is recruited to the cohesin complex by binding to a small domain on RAD21 (α-kleisin), located immediately distal to the N-terminal helices where RAD21 binds to SMC3 [22,23,46,56,57,78]. PDS5 promotes the establishment of sister chromatid cohesion by helping acetyltransferase (Eco1 in budding yeast; Eso1 in fission yeast; ESCO1 and ESCO2 in vertebrates) acetylate SMC3 during DNA replication at the S phase [26,28,78,79,80]. The binding of acetyltransferase to PDS5 is required to achieve the acetylation of SMC3. The HIM domain on the N-terminus of PDS5 and the PIM domain on fission yeast acetyltransferase Eso1 are responsible for the interaction of the two molecules [30]. However, except in the phyla *Schizosaccharomyces,* the PIM domain is not conserved [30]. Instead, PDS5 binds to the region containing amino residues 263–344 on human ESCO1, which is conserved among vertebrates [81]. The region on PDS5 that interacts with ESCO1 is on neither the N-terminus (1-200aa) nor the C-terminus [81], indicating the mechanisms of interactions between PDS5 and acetyltransferase in fission yeasts and vertebrates are different, although the functionality is conserved. The exact mechanisms whereby PDS5 promotes Eco1 in budding yeast and ESCO2 in vertebrates to acetylate SMC3 remain to be determined.

Once sister chromatid cohesion is established, the association of PDS5 to cohesin is required for the maintenance of cohesion through the G2/M phase, in which PDS5 prevents the de-acetylation of SMC3 [78] by the class I histone deacetylase Hos1 in yeast [25,82,83] and HDAC8 in mammalian cells [84]. Sororin competing with WAPL to bind to PDS5 is required for the maintenance of cohesion in metazoan [33,34,85,86]. Besides the PIM domain on sororin and WAPL that interacts with the HIM domain on PDS5, FGF motifs on sororin and WAPL are also important for their binding to PDS5 [33,34].

One FGF motif is present and conserved in sororin orthologs across different taxa of metazoan, whereas three FGF motifs are found in WAPL of vertebrates (not in yeast and fly WAPL) [87]. In sororin the FGF motif is close to the C-terminus of PIM, while in WAPL only one of the three FGF motifs is located proximately to PIM. The FGF motif is essential for sororin and WAPL to bind to PDS5, although there is controversy in which of the three FGF motifs on WAPL is more important for WAPL to interact with PDS5 [46,87].

Interestingly, yeasts do not have sororin [33], and yeast Wpl1 lacks most of the N-terminus found in vertebrate WAPL [87]. Considering the competitive binding of Sororin and WAPL to PDS5 and the antagonistic effects of sororin and WAPL in sister chromatid cohesion, it has been proposed that sororin and WAPL have functionally co-evolved in vertebrates [34].

WAPL and PDS5 form a complex that is responsible for unloading the cohesin complexes from chromosomes during the cell cycle, including G1, S, G2 and prophase in metazoan. It is important for the dynamics of cohesin associating with chromosomes during cell processes, such as gene transcription, DNA replication, and DNA damage repair. Depletion of either WAPL or PDS5 prolongs the binding of cohesins to chromosomes [32,88]. When the cell cycle progresses to the M phase, cohesion must be resolved in order for the sister chromatids to be segregated into two daughter cells. In yeast, the Wpl1-Pds5 cohesin unloading complex seems not to function on the removal of cohesin from sister chromatids at the M phase because all cohesins associated with chromosomes are cleaved by separase during the transition of metaphase to anaphase [89,90].

**Figure 3:**
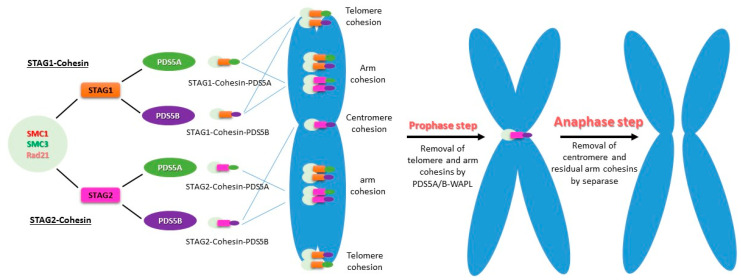
Stepwise cohesin removal from chromosomes in vertebrates. PDS5A and PDS5B bind to STAG1-cohesin or STAG2-cohesin, forming four different types of complexes, to execute their functions in the establishment, maintenance, and resolution of sister chromatid cohesion at S, G2, and M phases, respectively. All four PDS5-associated cohesins contribute to the chromosomal arm cohesion. The telomere regions contain PDS5A- and PDS5B-associated STAG1-cohesin specifically, whereas the centromeres have STAG2-cohesin-PDS5B only. At prophase, WAPL displaces phosphorylated sororin and binds to PDS5 to release the cohesins on the arm and telomere. At the transition of metaphase and anaphase, activated separase cleaves cohesin subunit Rad21, resulting in cohesin on the centromere and residual cohesin on the arm being released. Therefore, the sister chromatids are free to be segregated into two daughter cells.

However, sister chromatid cohesion in metazoan cells is resolved in two steps: the prophase step and the anaphase step [36,91]. There are two types of cohesin complexes, SMC1-SMC3-RAD21-STAG1 (STAG1-cohesin) and SMC1-SMC3-RAD21-STAG2 (STAG2-cohesin), in vertebrate mitotic cells. PDS5A and PDS5B can associate with either of the two cohesins, forming a total of four complexes (STAG1/2-cohesin-PDS5A/B) that contribute to the sister chromatid cohesion at different regions of the chromosome (Figure 3) [92,93]. All four complexes contribute to the chromosomal arm cohesion, whereas STAG1-cohesin-PDS5A and STAG1-cohesin-PDS5B are responsible for telomere cohesion. Only STAG2-cohesin-PDS5B is responsible for centromere cohesion [93]. 

In mitosis, sororin is phosphorylated at prophase and displaced by WAPL, which then binds to PDS5 to release most of the cohesin from the chromosomal arms [9,33,34,94,95]. The remaining cohesins from the chromosomal arms and all cohesins on centromeres, which are protected by shugoshin from the PDS5-WAPL mechanism (Sgol1, Sgol2), are destroyed by separase at the onset of anaphase [36,40,91]. Due to the spatial localization of PDS5A-cohesin and PDS5B-cohesin, PDS5A and PDS5B are removed together with their binding cohesins from chromosomal arms at the prophase, whereas centromere PDS5B-cohesin is released at the metaphase and anaphase transition [96]. It suggests that the specificity of telomeric cohesion is determined by STAG1-cohesin, whereas that of centromeric cohesion is determined by STAG2-cohesin and PDS5B.

### 3.2. Role in DNA Repair and Homologous Recombination

DNA damage that is caused by extracellular and intracellular DNA-damaging agents and events occurs frequently during the cell cycle. Two distinct mechanisms are used in eukaryote cells to repair DNA double-strand breaks (DSB): the non-homologous end-joining (NHEJ) pathway and the homologous recombination (HR) pathway. The NHEJ pathway connects the two free ends of damaged DNA, which often results in the loss of genetic information because it cannot recover the lost nucleotides or epigenetic modification at the break site. To restore the original sequence information, the HR pathway utilizes the sister chromatid as a template to faithfully repair the damaged DNA. Which pathway to use to repair DNA DSB depends on the phase of the cell cycle when the damage occurs [97]. If DNA damage happens at the G1 phase, it is repaired by the NHEJ pathway because no homologous chromosome can be used as a template. If DSB occurs at the S phase, both NHEJ and HR pathways are operational depending on the location where the DNA has been replicated or not. If DNA breaks at the G2-phase, the HR pathway is the predominant method of repair [98].

The function of cohesin in the repair of DSBs is conserved from yeast to humans [99,100,101,102,103]. Cohesin plays at least three important roles in DSB repair: transcription suppression at damage sites, DNA damage-induced cohesion (DI-cohesion), and checkpoint activation. In human cells, once DNA DSB is detected by DNA-damage sensors (ATM, ATR, MRE11, RAD50, NBS1, etc.) at S and G2/M phases, cohesins are recruited to re-enforce the cohesion genome-wide [5]. At the breaking site, cohesin complexes are recruited in a Mre11/Rad50-dependent manner [6] and cohesin subunits SMC1 and SMC3 are phosphorylated in an ATM- and NBS1-dependent fashion [104,105,106,107]. The DNA damage-induced cohesion not only enforces the sister chromatid cohesion, but also facilitates the recruitment of other checkpoint proteins, such as the mediator protein 53BP1 [108], to the DNA-break sites to activate the checkpoints. Cohesin is also required to suppress transcription at DNA DSB in the entire interphase [109]. Although there is no sister chromatid cohesion in the G1 phase because DNA replication has not yet occurred, cohesin is essential for the phosphorylation and activation of Chk2, suggesting that the roles of cohesin in checkpoint function and sister chromatid cohesion are independent [108]. Although cohesin has been found to activate DNA checkpoints in higher eukaryotes, this pathway has not been confirmed in yeast [3]. Besides cohesin itself, cohesin-regulating factors that function on cohesin loading, cohesion establishment, or cohesion maintenance also play important roles in repairing the damaged DNA [80,86,100,110,111,112,113,114]. As an associated protein, PDS5 plays a crucial role in repair of damaged DNA.

In *S. pombe,* Pds5 is important for DSB repair during meiotic recombination. *pds5* mutant cells are more sensitive to DNA damage than the wild type cells [115]. DSB repair is dependent on PDS5, as *pds5* null cells could not fully repair the damage [116]. In addition, Pds5 is responsible for pairing homologues and limiting chromosome compaction in meiosis [116]. The requirement of PDS5 for DSB repair is similar in other organisms. In the plant *Arabidopsis thaliana*, mutations in the *AtPDS5* gene caused dysfunctions in DNA-damage response and DSB repair, as well as a reduction of homologous recombination [117]. In *Drosophila*, PDS5 forming a complex with Brca2 mobilizes persisting meiotic DNA double-strand breaks to the nuclear envelope for repair [118].

Cohesin suppresses gene transcription at the region of the DNA-damage site, but depletion of PDS5B fails to suppress transcription, similar to other cohesin core subunits SMC3, RAD21, and STAG2, suggesting the function of cohesin in transcription suppression is disrupted when PDS5B is absent. Interestingly, depletion of PDS5A or STAG1 does not affect gene transcription [109], implying PDS5B and STAG2-cohesin play a critical role in transcription suppression at chromatin regions with damaged DNA. Mechanistic study indicates that PDS5B is recruited to DNA-damage sites before the HR mediator protein Rad51. Depletion of PDS5B reduces another HR mediator protein, PALB2, recruitment to the damage site, whereas knockdown of PALB2 does not affect accumulation of PDS5B on the damage site [76]. In an in vitro interaction assay using purified proteins from the sf9 protein expression system, PDS5B directly interacts with Rad51, PALB2, and BRCA2 [76]. In addition, PDS5B does not bind dsDNA but can bind diverse HR intermediates and preferentially binds displacement-loop (D-loop) structures over ssDNA, splayed arms (SA), and holiday junction (HJ) [76].

To repair the DSB, the dsDNA is required to be resected and the newly formed ssDNA is protected by replication protein A (RPA). Prior to strand invasion, RPA must be removed from the single strand tail to allow formation of the RAD51 filament to promote the presynaptic filament assembly. Such function is known to be accomplished by BRCA2 and PALB2 recombination mediators [119,120]. PDS5B can also displace RPA and facilitate RAD51 binding to ssDNA, as well as stimulate RAD51-mediated D-loop formation [76], suggesting that PDS5B, similar to PALB2 and BRCA2, functions as a mediator of HR, a fail-safe mechanism in case of a malfunction of the canonical PALB2/BRCA2 pathway.

Cohesin and its loading complex subunit NIPBL are involved in NHEJ during immunoglobulin (Ig) class switch recombination (CSR), whereas the unloading protein WAPL is not important in this process [121]. Interestingly, knockdown or knockout of PDS5B does not affect NHEJ during regular dsDNA-break repair or CSR [76], implying that PDS5B functions only in HR. Whether or not PDS5A plays a role in DNA DSB similar to PDS5B remains unclear. The study of the PDS5′s role in protecting the stalled replication forks indicates that both PDS5A and PDS5B can recruit fork-protective factors WRNIP1, RAD51 recombinase, and BRCA2 to protect the nascent DNA and keep the integrity of stalled replication forks [32]. Because those proteins also function in DNA DSB repair, it is likely that PDS5A and its functional paralog PDS5B may have a similar role in DSB repair.

### 3.3. Role in Gene Expression and Chromatin Architecture

Besides its main role in sister chromatid cohesion and DNA repair, cohesin also organizes interphase chromatin, regulating gene expression and chromatin architecture [122]. Depleting PDS5 causes loss of stability in cohesin-dependent loops, which are an important mechanism of gene regulation [88,123]. In *S. cerevisiae,* inactivation of PDS5 leads to an increase in the sizes of the DNA loops [123]. Mutations in *Pds5* in *Drosophila* also altered its regular gene-expression mechanism [124]. In the fruit fly, *Pds5 null* mutation increases the number of nicks on the edges of the wings by decreasing the expression of the *cut* gene, whereas *Pds5* N-terminal truncated mutation increases *cut* expression and decreases wing nicks [124]. Because cohesins serve as physical barriers to loop growth, removal of PDS5 relieves cohesin’s restrictions. Thus, PDS5-depleted cells have increased long-range contacts, as loops can expand for longer distances [123,125]. However, whether the effect of PDS5 on gene expression is direct or indirect through its functions on cohesin is not very well defined.

Eukaryotic genomes are spatially organized into compartments, topologically association domains (TAD), and loops to facilitate the chromosome-structure compaction and gene regulation [126,127,128]. Cohesin and CCCTC binding factor (CTCF) are the major players in defining TAD and loops [88,129,130,131,132,133,134,135,136]. CTCF, a zinc finger DNA-binding protein, functions in various aspects of gene regulation, such as serving as an enhancer-blocking transcriptional insulator [137,138] and facilitating enhancer–promoter interactions [139]. In mammalian genomes, approximately 90% of cohesin is localized to the binding sites for CTCF in chromosome arms [140,141]. It has been hypothesized that cohesin functions as a loop extruder factor, which partners with the CTCF that defines the directional boundaries of chromatin to generate chromatin loops [142,143], and accumulating evidence supports this loop-extruding hypothesis [88,128,136,144,145].

WAPL and PDS5 play crucial roles in forming TAD and loops via cohesin and CTCF. When WAPL is depleted, cohesin cannot be properly released from DNA, resulting in mild compaction of chromatin and accumulation of cohesin in axial structures (“vermicelli”) [39]. Knockdown of PDS5A or PDS5B alone has little effect on the chromatin’s compaction and cohesin’s localization, and co-depletion of WAPL with either PDS5A or PDS5B does not enhance the chromatin’s morphology or the vermicelli’s phenotype compared with the depletion of WAPL alone [88]. However, the severity of chromatin’s compaction and vermicelli increases with co-depletion of PDS5A and PDS5B [46,88], and even more when WAPL, PDS5A, and PDS5B are depleted simultaneously [88]. These findings suggest that PDS5 proteins regulate cohesin’s localization and chromatin’s compaction, and the functions of PDS5A and PDS5B in these processes are redundant [88].

WAPL and PDS5 form a cohesin-releasing complex that plays a crucial role in the dissociation of cohesin from chromatin [29,39,146,147,148,149]. The loop-extruding hypothesis predicts that depletion of WAPL and PDS5 leads to a longer association of cohesin with chromatin and larger chromatin loops, which is consistent with the findings of cohesin accumulated in vermicelli and compacted chromatin [39,46,88]. Further study shows that chromatin-interaction patterns are different between depletions of WAPL and PDS5, suggesting that WAPL functions through reducing residence time of cohesin on chromatin, whereas PDS5 functions through stopping loop extrusion once it recognizes the boundary element CTCF [88]. Thus, PDS5 probably regulates gene transcription via cohesin-modulated enhancer-promoter interaction.

## 4. Animal Models and Effects of PDS5 Proteins in Development

Two sets of PDS5A and PDS5B knockout mouse models have been reported [74,75,93] (Table 1). For easy discussion, they are called *Zhang’s model* and *Carretero’s model*, respectively. In Zhang’s model, PDS5A-deficient mice were generated using gene-trap with an insertion of the β-geo transgene in the second intron and disrupting the normal splicing of downstream of *Pds5A* exon 2 [75], whereas *Pds5B* knockout mice were developed by homologous recombination, in which the coding region of the second exon of *Pds5B* is replaced by a β-gal [74]. In Carretero’s model, conditional knockout mice of *Pds5A* and *Pds5B* were generated with loxp flanking exon 6 of *Pds5A* or exon 4-5 of *Pds5B*, respectively. Excisions of the targeted exons were achieved by crossing with mice ubiquitously expressing the Cre recombinase (CMV-Cre). Elimination of these exons leads to premature termination of translation and complete absence of the PDS5 protein [93].

In Zhang’s model, *Pds5A^+/^^−^*, *Pds5B^+/^^−^*, and *Pds5A^+/^^−^;Pds5B^+/^^−^* mice are viable and fertile [75] (Table 1). PDS5A null animals die perinatally and are smaller in size compared to the *Pds5A*-WT and heterozygous newborn pups. *Pds5A^−/^^−^* mice display cervical ribs and abnormal ossification patterns and also have a higher incidence of cleft palate than do the heterozygous counterparts [75]. Deficiency in PDS5A in general led to growth retardation and cleft palate and displayed an atypical pattern of skeleton. PDS5A-deficient animals also had defects in renal development, which could contribute to their perinatal death. However, PDS5A null animals have usual sympathetic neuronal projections, which shows that PDS5A is not required for the development of the central nervous system [75].

Approximately 75% of *Pds5B^−/^^−^* mice were born alive, but they all died soon after birth, not surviving after P1. PDS5B deficiency in newborns led to growth retardations, abnormal skeleton, and cleft palate. However, in contrast to *Pds5A^−/^^−^* mice, *Pds5B* mutants displayed abnormality in the development of the peripheral nervous system and in sympathetic innervation [74,75].

The abnormalities between the *Pds5A* and *Pds5B* knockout mice are very similar to each other, which indicates PDS5A and PDS5B can play redundant roles in embryonic development, and likely *PDS5A* can compensate for loss of *PDS5B* and vice versa [75]. Both homologues are required for cardiac development, and their lack causes congenital heart defects. Both are also important for palatogenesis and skeletal patterning [74,75]. However, PDS5 deficiency did not affect mice’s brains, showing that their role in the cohesin complex does not affect the development of the CNS [74,75].

Compound heterozygotes animals were obtained by crossing *Pds5A^+/^^−^* with *Pds5B^+/^^−^* mice, which were then crossed to generate double homozygous or compound homozygous-heterozygous mice [75]. Depletion of both PDS5 proteins (double homozygous mice) led to early embryonic death at E9.5. Mice lacking one of the two PDS5 proteins and heterozygous allele of the other (homozygous-heterozygous mice) had growth retardation, and this depletion led to midgestational lethality in E11.5 and E12.5 for *Pds5A^+/−^;Pds5B^−/−^* and *Pds5A^−/^^−^;Pds5B^+/^^−^* mice, respectively. Both *Pds5A^−/^^−^;Pds5B^+/^^−^* and *Pds5A^+/^^−^;Pds5B^−/^^−^* mice had many cardiac dysfunctions, such as truncus arteriosus, a single joint for the atrioventricular canal, a thin compact ventricular myocardium, and dilated atria [75]. However, *Pds5A^−/^^−^;Pds5B^+/^^−^* embryos had lens agenesis more often, whereas *Pds5A^+/^^−^;Pds5B^−/^^−^* animals displayed hypoplastic lenses. Because expression of *Pds5A* is much higher than that of *Pds5B* in head surface ectoderm at E9.5, a stage when the lens is beginning to form, it suggests that formation of lenses depends on PDS5A. Both PDS5 proteins are important for development of the enteric nervous system, but lack of PDS5B leads to more severe outcomes than deficiency of PDS5A [75]. Thus, different dosages of the genes have an important outcome in mice, as they display distinct phenotypes. Loss of either PDS5A or PDS5B leads to lethality shortly after birth, whereas lack of three alleles causes embryonic death. As it is not possible to generate *Pds5A;Pds5B* double homozygous, it is implicit that PDS5 function is required for early embryogenesis [75].

Interestingly, the findings in embryonic defects of PDS5A-null and PDS5B-null mice in Zhang’s model are not consistent with those in Carretero’s model (Table 1). Carretero et al. observed that *Pds5A* and *Pds5B* are non-redundant, and both are essential to fulfill embryonic development because knockout of either *Pds5A* or *Pds5B* caused embryonic lethality [93]. In addition, analysis of mouse embryonic fibroblasts (MEFs) lacking PDS5A, PDS5B, or both proteins revealed that PDS5A and PDS5B have different functions in sister chromatid cohesion. Both PDS5A and PDS5B contribute to telomere and arm cohesion, whereas centromeric cohesion relies specifically on PDS5B [93]. Moreover, MEFs and embryonic hepatocytes lacking PDS5B display loss of sister chromatid cohesion and aneuploidy [93], but this finding is contradictory to the result of Zhang’s model, in which MEFs lacking PDS5B show normal sister chromatid cohesion [74]. More detailed studies are required to resolve these discrepancies between these two models.

## 5. Cohesinopathies

Mutations in genes of the cohesin complex and its regulators are important genetic factors that can contribute to rare human diseases with developmental phenotypes, which is collectively known as *cohesinopathies* [84,151,152,153,154,155,156]. Mutations in cohesin and its associate proteins are often linked to the development of Cornelia de Lange syndrome (CdLS). The syndrome is rare and known by the phenotypes including craniofacial features (low anterior hairline, synophrys, arched thick eyebrows, long eyelashes, thin upper vermilion, and downturned corners of the mouth, etc.), growth retardation, intellectual disability, myopia, congenital heart defects, limb and organ defects, abnormal skeleton pattern, cleft palate. [156,157]. Some patients have abnormal behaviors, such as autism spectrum disorder, self-injury, and stereotypic movements [156]. Some characterized molecular features in individuals with CdLS include mutations in the subunit of cohesin-loading complex, NIPBL, and in cohesin-structural subunits, SMC1A and SMC3. At least half of the CdLS cases have loss-of-function mutations in NIPBL, which implies that defects in cohesin are highly associated with CdLS [152,153,155,156,158].

PDS5A and PDS5B knockout mice display most of the characteristics of CdLS [74,75]. However, cases of CdLS that are caused by PDS5 mutations are rare. A study sequencing the genes of PDS5B and PDS5A from 114 CdLS patients who had no mutations in NIPBL, SMC1A, and SMC3 identified a missense mutation (R1292Q) on PDS5B from one patient [75]. This missense mutation occurred at the invariant core sequence (GRP) of the AT-hook DNA binding motif (Figure 2). Further study revealed that this mutation was inherited from the proband’s father, who had a consanguineous marriage. In this family, three of the four children have CdLS; two of the three children with CdLS were confirmed to bear the R1292Q mutation and whether the third one had the PDS5B mutation was unknown because he was deceased before this mutation was identified. The father and the fourth child do not show CdLS, although they have the R1292Q mutation on PDS5B. The mother does not have this mutation and CdLS. It suggests that R1292Q mutation alone does not cause CdLS. Interestingly, besides the mutated PDS5B, sequencing analysis showed that the other allele of PDS5B in the two affected siblings was the same, which is different from the PDS5B allele that the unaffected sibling inherited [75]. It implies that R1292Q is transmitted in a recessive manner in this family, and it required another hit for the mutated PDS5B to show CdLS in a person. The secret might hide in the maternal chromosomes, which remains to be revealed.

## 6. Cancer

Because PDS5 plays important roles in chromatin architecture and gene regulation, it is not surprising that dysregulation of PDS5 expression (deletion, up-regulation, down-regulation, mutation, etc.) could link to initiation and development of tumors. Abnormal expression of PDS5 has been found in a variety of cancers, such as prostate, cervical, head and neck, and esophageal [73,159,160,161,162,163]. PDS5B has been suggested as a tumor suppressor because its expression is reduced or lost in many cancer types [164]. PDS5B coordinates the differentiation of stem cells, and knockdown of PDS5B causes the failure of differentiation and results in immature proliferative cells that are similar to the cancer-initiation cells [165].

Immunohistochemistry analysis indicates that nearly 47% of breast tumors tested had reduced expression of PDS5B and the frequency of low PDS5B expression is significantly correlated with histological grade (i.e., the higher the grade of cancers, the lower the expression of PDS5B) [166]. Low expression of PDS5B is also correlated with the ER-negative tumors (64.7%), especially with basal-like/triple-negative tumors (67%). Those findings were confirmed by another study using microarray data, indicating that reduced expression of PDS5B in tumors is significantly correlated with the basal-like phenotype, when compared with HER2-positive and luminal subtypes [166]. A common mechanism that regulates gene expression is epigenetic modification. The methylation of CpG on PDS5B promoter is found to be inversely correlated to the expression of mRNA [166], suggesting that promoter methylation may be a mechanism for PDS5B silencing in tumors with normal copy numbers. Because PDS5 is essential for the HR during DNA repair, loss of expression could sensitize tumor cells to agents that cause DNA damage. When patients with ER-negative breast cancer were treated with adjuvant anthracycline-based chemotherapy, those with low expression of PDS5B in their tumors had a statistically significant longer metastasis-free survival or disease-free survival compared to those with high expression of PDS5B [166]. The association of reduced expression of PDS5B with increase of survival rates is also observed in patients with pancreatic cancer [167]. Because anthracycline is a DNA-intercalating agent and causes DNA damage, the DNA-repair mechanism is probably weakened in tumor cells with low expression of PDS5B. The correlation between low expression of PDS5B levels and better survival rates is also found in patients with ovarian cancer [76].

A mechanism whereby PDS5B depletion promotes proliferation of cancer cells is possible through the IL-6/STAT3/cyclin D axis [168]. Depletion of PDS5B in cancer cells induces secretion of interleukin-6 (IL-6), which binds to its receptor and in turn activates intracellular STAT3. The activated STAT3 (pSTAT3) dimerizes and are translocated to the nucleus where it, together with other transcription factors, binds to the promoter of cyclin D1 and enhances the expression of cyclin D1 [168]. Cyclin D1 in turn binds to Cdk4/6, which drives the progression of cell cycle from G1 to S phase. This information suggests targeting IL-6/STAT3/cyclin D axis in cancers with low PDS5B might provide a novel therapeutic approach [168].

Another mechanism to reduce PDS5 by tumor cells is through microRNA. miRNA-223 is overexpressed in numerous cancers and is proposed to be used as a diagnostic/prognostic marker in cancers such as pediatric lymphoblastic T cell lymphoma, lung cancer, gastroesophageal adenocarcinoma, oral cancer, and pancreatic cancer [169,170,171,172,173,174,175,176,177]. Recently, miR-223 was found to target PDS5B mRNA [178]. miR-223 causes the reduction of PDS5B protein levels and promotes the growth of pancreatic cancer cells, whereas increase of PDS5B by overexpressing PDS5B or miRNA-223 inhibitor inhibits cancer cell growth [178].

Gene mutations are common in tumors with loss of PDS5 functions. Loss of PDS5B was found in gastric and colorectal cancers, which commonly result from frameshift mutations or high microsatellite instability [164]. Although the mechanisms that lead to the reduction/loss of PDS5 expression may be different for different tumors, the goal seems to be the same (i.e., tumor cells try to eliminate the suppressing property of PDS5 for their growth). This finding creates a caveat for the tumor cells that are vulnerable to cancer treatments, such as radiation and chemotherapy. As a result, low expression of PDS5B levels is correlated with better survival in patients with breast and ovarian cancer [76,166].

Currently most studies are focused on the status of PDS5B on tumors. PDS5A may also play a critical role in initiation and development of cancer. A study of the paired sets of human tumor and normal tissues revealed that the PDS5A mRNA and protein levels were significantly low in primary tumor tissues as compared to the corresponding normal breast and kidney tissues. Overexpression of PDS5A in Cos-1 monkey kidney cells and MDA-MB 231 human breast cancer cells results in accumulation of G1-phase cells and increase of cell death [179]. Interestingly, compared with matched normal tissues, up-regulation of PDS5A in some tumors has also been reported [180,181]. PDS5A is significantly overexpressed in human gliomas, and its overexpression is correlated positively with the tumor grade [180]. PDS5A is reported to be up-regulated in esophagus, stomach, and liver tumors tested in each paired set of normal and tumor tissues. Overexpression of PDS5A in 293T and three nasopharyngeal cell lines promotes cell proliferation and clone formation, whereas knockdown of PDS5A inhibits cell growth [181]. The mechanism by which PDS5A overexpression promotes tumor growth remains to be revealed.

PDS5A and PDS5B have their unique and redundant functions in the cell cycle, and depletion of both proteins will cause severe defects or cell death [75,93,96,182]. The redundant function of PDS5A and PDS5B provides a possibility to develop a therapeutic approach by using a synthetic lethal strategy. When a tumor lacks PDS5B, depletion of PDS5A is expected to inhibit the tumor’s growth, and vice versa. Similar strategies are currently being explored for STAG1 and STAG2, two paralogs of cohesin subunits, and shows the depletion of STAG1 selectively kills SATG2-deficient cancer cells [183,184,185,186].

## 7. Conclusions

PDS5 is a versatile protein regulating a variety of cell processes by partnering with different proteins such as acetyltransferase, WAPL, sororin, Hrk1, and BRCA2. Most of the roles that PDS5 plays are plausibly via regulating the association of cohesin with chromosomes. Whether or not PDS5 can function independent of cohesin remains unclear. In vertebrates, the roles of PDS5 are extended by harboring two homologs, PDS5A and PDS5B. They play redundant and specific functions in temporal and spatial manners. Both cell lines and animal models with knockout or overexpression of PDS5A/B are critical tools in defining their redundant and unique functions in cell growth and development. Identification of disease-specific mutations is both important for the mechanistic study and therapy.

## Figures and Tables

**Table 1:** Comparison of PDS5A and PDS5B functions in mouse models and cell lines.

	Genotype/Phenotype/Functions	Note	References
Mouse Models	*Pds5A^+/−^*: viable, fertile Pds*5B^+/−^*: viable, fertile		[74,75,93]
	*Pds5A^−/−^*: embryonic lethality ^a^/perinatal lethality ^b^ *Pds5B^−/−^*: embryonic lethality ^a^/perinatal lethality ^b^	PDS5A null has more severe effect on embryonic development than does PDS5B null.^a^	[75,93]
	*Pds5A^+/−^;Pds5B^+/−^*: viable, fertile		[75]
	*Pds5A^−/−^;Pds5B^+/−^*: embryonic lethality *Pds5A^+/−^;Pds5B^−/−^*: embryonic lethality		[75]
	Common developmental abnormalities found in *Pds5A^−/−^* and *Pds5B^−/−^* mice: including cleft palate, skeletal patterning defects, growth retardation, congenital heart defects and delayed migration of enteric neuron precursors. Specific developmental abnormalities found in *Pds5A^−/−^* mice: renal agenesis. *Pds5B^−^^/−^* mice: abnormalities in the superior cervical ganglia (SCG) and its projections to target organs; severe depletion of primordial germ cells in the testes and ovaries.	PDS5A is not required for meiosis or primordial germ cell development.	[74,75]
MEF Cells	*Pds5A^−/−^* MEFs grow slow than WT cells. *Pds5B^−/−^* MEFs grow slow than WT cells.	Pds5A null has more pronounced defect on cell proliferate than does PDS5B null.	[93]
	*Pds5A^−/−^* MEFs increase chromosome-bound cohesin. *Pds5B^−/−^* MEFs increase chromosome-bound cohesin.	Suggesting both PDSA and PDS5B are required in cohesin unloading.	[93]
	PDS5A: required for SMC3 acetylation. PDS5B: required for SMC3 acetylation.		[93]
	PDS5A: required for the recruitment of WAPL and sororin. PDS5B: required for the recruitment of WAPL and sororin.		[93]
	PDS5A: chromosome arm and telomere cohesion. PDS5B: chromosome arm and telomere cohesion		[93]
	PDS5B functions in centromeric cohesion and promotes ESCO2, sororin, Aurora B to localize to pericentric heterochromatin (PCH)	PDS5A has no role in centromeric cohesion.	[93]
	PDS5B null: aneuploidy		[93]
	PDS5A: replication fork protection. PDS5B: replication fork protection.		[32]
	*Pds5A^−/−^;Pds5B^−/−^*: decrease cohesin mobility in G0-arrested MEFs. Also observed in HeLa cells [46,88]	PDS5A null or PDS5B null barely affects cohesin dynamics.	[32]
	*Pds5A^−/−^;Pds5B^−/−^*: reduce replication fork velocity compared with their WT counterparts. Also confirmed in HeLa cells.	PDS5A null or PDS5B null does not affect the fork velocity. Reducing cohesin restores the fork velocity in Hela cells with PDS5 knock down.	[32]
Cell Lines	PDS5A and PDS5B recruit and/or stabilize the fork protection complex (Brca2, Rad51, WRNIP1 etc.) at stalled forks possibly via cohesin in HeLa.		[32]
	PDS5A and PDS5B protect nascent DNA strands from MRE11 Degradation.		[32,150]
	PDS5A and PDS5B depletion facilitates DNA compaction in HeLa.	Both PDS5A and PDS5B regulate cohesin dynamics and chromatin compaction.	Ouyang, 2016 1979/id; Wutz, 2017 1982/id
	PDS5A and PDS5B interact with sororin and WAPL		[33,146,147]
	PDS5A: cannot repress transcription during DNA damage PDS5B: repress transcription during DNA damage		[109]

^a^ [93], ^b^ [75].

## Data Availability

Not applicable.

## References

[1] Covo S., Westmoreland J.W., Gordenin D.A., Resnick M.A. (2010). Cohesin Is limiting for the suppression of DNA damage-induced recombination between homologous chromosomes. PLoS Genet..

[2] Dorsett D. (2011). Cohesin: Genomic insights into controlling gene transcription and development. Curr. Opin. Genet. Dev..

[3] Dorsett D., Strom L. (2012). The ancient and evolving roles of cohesin in gene expression and DNA repair. Curr. Biol..

[4] Fay A., Misulovin Z., Li J., Schaaf C.A., Gause M., Gilmour D.S., Dorsett D. (2011). Cohesin selectively binds and regulates genes with paused RNA polymerase. Curr. Biol..

[5] Kim B.J., Li Y., Zhang J., Xi Y., Li Y., Yang T., Jung S.Y., Pan X., Chen R., Li W. (2010). Genome-wide reinforcement of cohesin binding at pre-existing cohesin sites in response to ionizing radiation in human cells. J. Biol. Chem..

[6] Kim J.S., Krasieva T.B., LaMorte V., Taylor A.M., Yokomori K. (2002). Specific recruitment of human cohesin to laser-induced DNA damage. J. Biol. Chem..

[7] Klein F., Mahr P., Galova M., Buonomo S.B., Michaelis C., Nairz K., Nasmyth K. (1999). A central role for cohesins in sister chromatid cohesion, formation of axial elements, and recombination during yeast meiosis. Cell.

[8] Michaelis C., Ciosk R., Nasmyth K. (1997). Cohesins: Chromosomal proteins that prevent premature separation of sister chromatids. Cell.

[9] Zhang N., Pati D., iConcept Press (2014). Road to cancer via cohesin deregulation. Oncology-Theory & Practice.

[10] Nakamura A., Arai H., Fujita N. (2009). Centrosomal Aki1 and cohesin function in separase-regulated centriole disengagement. J. Cell Biol..

[11] Darwiche N., Freeman L.A., Strunnikov A. (1999). Characterization of the components of the putative mammalian sister chromatid cohesion complex. Gene.

[12] Guacci V., Koshland D., Strunnikov A. (1997). A direct link between sister chromatid cohesion and chromosome condensation revealed through the analysis of MCD1 in S. cerevisiae. Cell.

[13] Vass S., Cotterill S., Valdeolmillos A.M., Barbero J.L., Lin E., Warren W.D., Heck M.M. (2003). Depletion of drad21/scc1 in Drosophila cells leads to instability of the cohesin complex and disruption of mitotic progression. Curr. Biol..

[14] Haering C.H., Lowe J., Hochwagen A., Nasmyth K. (2002). Molecular architecture of SMC proteins and the yeast cohesin complex. Mol. Cell.

[15] Anderson D.E., Losada A., Erickson H.P., Hirano T. (2002). Condensin and cohesin display different arm conformations with characteristic hinge angles. J. Cell Biol..

[16] Lowe J., Cordell S.C., van den E.F. (2001). Crystal structure of the SMC head domain: An ABC ATPase with 900 residues antiparallel coiled-coil inserted. J. Mol. Biol.

[17] Arumugam P., Nishino T., Haering C.H., Gruber S., Nasmyth K. (2006). Cohesin’s ATPase activity is stimulated by the C-terminal Winged-Helix domain of its kleisin subunit. Curr. Biol..

[18] Gligoris T.G., Scheinost J.C., Burmann F., Petela N., Chan K.L., Uluocak P., Beckouet F., Gruber S., Nasmyth K., Lowe J. (2014). Closing the cohesin ring: Structure and function of its Smc3-kleisin interface. Science.

[19] Chan K.L., Roig M.B., Hu B., Beckouet F., Metson J., Nasmyth K. (2012). Cohesin’s DNA exit gate is distinct from its entrance gate and is regulated by acetylation. Cell.

[20] Hara K., Zheng G., Qu Q., Liu H., Ouyang Z., Chen Z., Tomchick D.R., Yu H. (2014). Structure of cohesin subcomplex pinpoints direct shugoshin-Wapl antagonism in centromeric cohesion. Nat. Struct. Mol. Biol..

[21] Roig M.B., Lowe J., Chan K.L., Beckouet F., Metson J., Nasmyth K. (2014). Structure and function of cohesin’s Scc3/SA regulatory subunit. FEBS Lett..

[22] Rowland B.D., Roig M.B., Nishino T., Kurze A., Uluocak P., Mishra A., Beckouet F., Underwood P., Metson J., Imre R. (2009). Building sister chromatid cohesion: smc3 acetylation counteracts an antiestablishment activity. Mol. Cell.

[23] Sutani T., Kawaguchi T., Kanno R., Itoh T., Shirahige K. (2009). Budding yeast Wpl1(Rad61)-Pds5 complex counteracts sister chromatid cohesion-establishing reaction. Curr. Biol..

[24] Rankin S. (2015). Complex elaboration: Making sense of meiotic cohesin dynamics. FEBS J..

[25] Beckouet F., Hu B., Roig M.B., Sutani T., Komata M., Uluocak P., Katis V.L., Shirahige K., Nasmyth K. (2010). An Smc3 acetylation cycle is essential for establishment of sister chromatid cohesion. Mol. Cell.

[26] Ben-Shahar T.R., Heeger S., Lehane C., East P., Flynn H., Skehel M., Uhlmann F. (2008). Eco1-dependent cohesin acetylation during establishment of sister chromatid cohesion. Science.

[27] Hou F., Zou H. (2005). Two human orthologues of Eco1/Ctf7 acetyltransferases are both required for proper sister-chromatid cohesion. Mol. Biol. Cell.

[28] Zhang J., Shi X., Li Y., Kim B.J., Jia J., Huang Z., Yang T., Fu X., Jung S.Y., Wang Y. (2008). Acetylation of Smc3 by Eco1 is required for S phase sister chromatid cohesion in both human and yeast. Mol. Cell.

[29] Gause M., Misulovin Z., Bilyeu A., Dorsett D. (2010). Dosage-sensitive regulation of cohesin chromosome binding and dynamics by Nipped-B, Pds5, and Wapl. Mol. Cell Biol..

[30] Goto Y., Yamagishi Y., Shintomi-Kawamura M., Abe M., Tanno Y., Watanabe Y. (2017). Pds5 Regulates Sister-Chromatid Cohesion and Chromosome Bi-orientation through a Conserved Protein Interaction Module. Curr. Biol..

[31] Losada A., Yokochi T., Hirano T. (2005). Functional contribution of Pds5 to cohesin-mediated cohesion in human cells and Xenopus egg extracts. J. Cell Sci..

[32] Morales C., Ruiz-Torres M., Rodriguez-Acebes S., Lafarga V., Rodriguez-Corsino M., Megias D., Cisneros D.A., Peters J.M., Mendez J., Losada A. (2020). PDS5 proteins are required for proper cohesin dynamics and participate in replication fork protection. J. Biol. Chem..

[33] Nishiyama T., Ladurner R., Schmitz J., Kreidl E., Schleiffer A., Bhaskara V., Bando M., Shirahige K., Hyman A.A., Mechtler K. (2010). Sororin mediates sister chromatid cohesion by antagonizing Wapl. Cell.

[34] Zhang N., Pati D. (2012). Sororin is a master regulator of sister chromatid cohesion and separation. Cell Cycle.

[35] Gimenez-Abian J.F., Sumara I., Hirota T., Hauf S., Gerlich D., de la T.C., Ellenberg J., Peters J.M. (2004). Regulation of sister chromatid cohesion between chromosome arms. Curr. Biol..

[36] Hauf S., Waizenegger I.C., Peters J.M. (2001). Cohesin cleavage by separase required for anaphase and cytokinesis in human cells. Science.

[37] Hauf S., Roitinger E., Koch B., Dittrich C.M., Mechtler K., Peters J.M. (2005). Dissociation of Cohesin from Chromosome Arms and Loss of Arm Cohesion during Early Mitosis Depends on Phosphorylation of SA2. PLoS Biol..

[38] Nakajima M., Kumada K., Hatakeyama K., Noda T., Peters J.M., Hirota T. (2007). The complete removal of cohesin from chromosome arms depends on separase. J. Cell Sci..

[39] Tedeschi A., Wutz G., Huet S., Jaritz M., Wuensche A., Schirghuber E., Davidson I.F., Tang W., Cisneros D.A., Bhaskara V. (2013). Wapl is an essential regulator of chromatin structure and chromosome segregation. Nature.

[40] Zhang N., Pati D. (2017). Biology and insights into the role of cohesin protease separase in human malignancies. Biol. Rev. Camb. Philos. Soc..

[41] Akins R.A., Lambowitz A.M. (1985). General method for cloning Neurospora crassa nuclear genes by complementation of mutants. Mol. Cell Biol..

[42] Denison S.H., Kafer E., May G.S. (1993). Mutation in the bimD gene of Aspergillus nidulans confers a conditional mitotic block and sensitivity to DNA damaging agents. Genetics.

[43] Hartman T., Stead K., Koshland D., Guacci V. (2000). Pds5p is an essential chromosomal protein required for both sister chromatid cohesion and condensation in Saccharomyces cerevisiae. J. Cell Biol..

[44] Panizza S., Tanaka T., Hochwagen A., Eisenhaber F., Nasmyth K. (2000). Pds5 cooperates with cohesin in maintaining sister chromatid cohesion. Curr. Biol..

[45] van Heemst D., James F., Poggeler S., Berteaux-Lecellier V., Zickler D. (1999). Spo76p is a conserved chromosome morphogenesis protein that links the mitotic and meiotic programs. Cell.

[46] Ouyang Z., Zheng G., Tomchick D.R., Luo X., Yu H. (2016). Structural Basis and IP6 Requirement for Pds5-Dependent Cohesin Dynamics. Mol. Cell.

[47] Neuwald A.F., Hirano T. (2000). HEAT repeats associated with condensins, cohesins, and other complexes involved in chromosome-related functions. Genome Res..

[48] Andrade M.A., Bork P. (1995). HEAT repeats in the Huntington’s disease protein. Nat. Genet..

[49] Chao W.C., Murayama Y., Munoz S., Jones A.W., Wade B.O., Purkiss A.G., Hu X.W., Borg A., Snijders A.P., Uhlmann F. (2017). Structure of the cohesin loader Scc2. Nat. Commun..

[50] Kikuchi S., Borek D.M., Otwinowski Z., Tomchick D.R., Yu H. (2016). Crystal structure of the cohesin loader Scc2 and insight into cohesinopathy. Proc. Natl. Acad. Sci. USA.

[51] Li Y., Muir K.W., Bowler M.W., Metz J., Haering C.H., Panne D. (2018). Structural basis for Scc3-dependent cohesin recruitment to chromatin. Elife.

[52] Chatterjee A., Zakian S., Hu X.W., Singleton M.R. (2013). Structural insights into the regulation of cohesion establishment by Wpl1. EMBO J..

[53] Ouyang Z., Zheng G., Song J., Borek D.M., Otwinowski Z., Brautigam C.A., Tomchick D.R., Rankin S., Yu H. (2013). Structure of the human cohesin inhibitor Wapl. Proc. Natl. Acad. Sci. USA.

[54] Boland A., Martin T.G., Zhang Z., Yang J., Bai X.C., Chang L., Scheres S.H., Barford D. (2017). Cryo-EM structure of a metazoan separase-securin complex at near-atomic resolution. Nat. Struct. Mol. Biol..

[55] Viadiu H., Stemmann O., Kirschner M.W., Walz T. (2005). Domain structure of separase and its binding to securin as determined by EM. Nat. Struct. Mol. Biol..

[56] Lee B.G., Roig M.B., Jansma M., Petela N., Metson J., Nasmyth K., Lowe J. (2016). Crystal Structure of the Cohesin Gatekeeper Pds5 and in Complex with Kleisin Scc1. Cell Rep..

[57] Muir K.W., Kschonsak M., Li Y., Metz J., Haering C.H., Panne D. (2016). Structure of the Pds5-Scc1 Complex and Implications for Cohesin Function. Cell Rep..

[58] Wu F.M., Nguyen J.V., Rankin S. (2010). A conserved motif at the C-terminus of sororin is required for sister chromatid cohesion. J. Biol. Chem..

[59] Montpetit B., Thomsen N.D., Helmke K.J., Seeliger M.A., Berger J.M., Weis K. (2011). A conserved mechanism of DEAD-box ATPase activation by nucleoporins and InsP6 in mRNA export. Nature.

[60] Sheard L.B., Tan X., Mao H., Withers J., Ben-Nissan G., Hinds T.R., Kobayashi Y., Hsu F.F., Sharon M., Browse J. (2010). Jasmonate perception by inositol-phosphate-potentiated COI1-JAZ co-receptor. Nature.

[61] Macbeth M.R., Schubert H.L., Vandemark A.P., Lingam A.T., Hill C.P., Bass B.L. (2005). Inositol hexakisphosphate is bound in the ADAR2 core and required for RNA editing. Science.

[62] Traven A., Heierhorst J. (2005). SQ/TQ cluster domains: Concentrated ATM/ATR kinase phosphorylation site regions in DNA-damage-response proteins. Bioessays.

[63] Sardon T., Pache R.A., Stein A., Molina H., Vernos I., Aloy P. (2010). Uncovering new substrates for Aurora A kinase. EMBO Rep..

[64] Ferrari S., Marin O., Pagano M.A., Meggio F., Hess D., El-Shemerly M., Krystyniak A., Pinna L.A. (2005). Aurora-A site specificity: A study with synthetic peptide substrates. Biochem. J..

[65] Meraldi P., Honda R., Nigg E.A. (2004). Aurora kinases link chromosome segregation and cell division to cancer susceptibility. Curr. Opin. Genet. Dev..

[66] Ubersax J.A., Woodbury E.L., Quang P.N., Paraz M., Blethrow J.D., Shah K., Shokat K.M., Morgan D.O. (2003). Targets of the cyclin-dependent kinase Cdk1. Nature.

[67] O’Neill T., Giarratani L., Chen P., Iyer L., Lee C.H., Bobiak M., Kanai F., Zhou B.B., Chung J.H., Rathbun G.A. (2002). Determination of substrate motifs for human Chk1 and hCds1/Chk2 by the oriented peptide library approach. J. Biol. Chem..

[68] Liu Z., Ren J., Cao J., He J., Yao X., Jin C., Xue Y. (2013). Systematic analysis of the Plk-mediated phosphoregulation in eukaryotes. Brief. Bioinform..

[69] Aravind L., Landsman D. (1998). AT-hook motifs identified in a wide variety of DNA-binding proteins. Nucleic Acids Res..

[70] Kosugi S., Hasebe M., Tomita M., Yanagawa H. (2009). Systematic identification of cell cycle-dependent yeast nucleocytoplasmic shuttling proteins by prediction of composite motifs. Proc. Natl. Acad. Sci. USA.

[71] Sievers F., Wilm A., Dineen D., Gibson T.J., Karplus K., Li W., Lopez R., McWilliam H., Remmert M., Soding J. (2011). Fast, scalable generation of high-quality protein multiple sequence alignments using Clustal Omega. Mol. Syst. Biol..

[72] Elia A.E., Boardman A.P., Wang D.C., Huttlin E.L., Everley R.A., Dephoure N., Zhou C., Koren I., Gygi S.P., Elledge S.J. (2015). Quantitative Proteomic Atlas of Ubiquitination and Acetylation in the DNA Damage Response. Mol. Cell.

[73] Maffini M., Denes V., Sonnenschein C., Soto A., Geck P. (2008). APRIN is a unique Pds5 paralog with features of a chromatin regulator in hormonal differentiation. J. Steroid Biochem. Mol. Biol..

[74] Zhang B., Jain S., Song H., Fu M., Heuckeroth R.O., Erlich J.M., Jay P.Y., Milbrandt J. (2007). Mice lacking sister chromatid cohesion protein PDS5B exhibit developmental abnormalities reminiscent of Cornelia de Lange syndrome. Development.

[75] Zhang B., Chang J., Fu M., Huang J., Kashyap R., Salavaggione E., Jain S., Kulkarni S., Deardorff M.A., Uzielli M.L. (2009). Dosage effects of cohesin regulatory factor PDS5 on mammalian development: Implications for cohesinopathies. PLoS ONE.

[76] Couturier A.M., Fleury H., Patenaude A.M., Bentley V.L., Rodrigue A., Coulombe Y., Niraj J., Pauty J., Berman J.N., Dellaire G. (2016). Roles for APRIN (PDS5B) in homologous recombination and in ovarian cancer prediction. Nucleic Acids Res..

[77] Reeves R. (2000). Structure and function of the HMGI(Y) family of architectural transcription factors. Environ. Health Perspect..

[78] Chan K.L., Gligoris T., Upcher W., Kato Y., Shirahige K., Nasmyth K., Beckouet F. (2013). Pds5 promotes and protects cohesin acetylation. Proc. Natl. Acad. Sci. USA.

[79] Tanaka K., Hao Z., Kai M., Okayama H. (2001). Establishment and maintenance of sister chromatid cohesion in fission yeast by a unique mechanism. EMBO J..

[80] Unal E., Heidinger-Pauli J.M., Kim W., Guacci V., Onn I., Gygi S.P., Koshland D.E. (2008). A molecular determinant for the establishment of sister chromatid cohesion. Science.

[81] Minamino M., Ishibashi M., Nakato R., Akiyama K., Tanaka H., Kato Y., Negishi L., Hirota T., Sutani T., Bando M. (2015). Esco1 Acetylates Cohesin via a Mechanism Different from That of Esco2. Curr. Biol..

[82] Borges V., Lehane C., Lopez-Serra L., Flynn H., Skehel M., Rolef Ben-Shahar T., Uhlmann F. (2010). Hos1 deacetylates Smc3 to close the cohesin acetylation cycle. Mol. Cell.

[83] Xiong B., Lu S., Gerton J.L. (2010). Hos1 is a lysine deacetylase for the Smc3 subunit of cohesin. Curr. Biol..

[84] Deardorff M.A., Bando M., Nakato R., Watrin E., Itoh T., Minamino M., Saitoh K., Komata M., Katou Y., Clark D. (2012). HDAC8 mutations in Cornelia de Lange syndrome affect the cohesin acetylation cycle. Nature.

[85] Lafont A.L., Song J., Rankin S. (2010). Sororin cooperates with the acetyltransferase Eco2 to ensure DNA replication-dependent sister chromatid cohesion. Proc. Natl. Acad. Sci. USA.

[86] Schmitz J., Watrin E., Lenart P., Mechtler K., Peters J.M. (2007). Sororin is required for stable binding of cohesin to chromatin and for sister chromatid cohesion in interphase. Curr Biol..

[87] Shintomi K., Hirano T. (2009). Releasing cohesin from chromosome arms in early mitosis: Opposing actions of Wapl-Pds5 and Sgo1. Genes Dev..

[88] Wutz G., Varnai C., Nagasaka K., Cisneros D.A., Stocsits R.R., Tang W., Schoenfelder S., Jessberger G., Muhar M., Hossain M.J. (2017). Topologically associating domains and chromatin loops depend on cohesin and are regulated by CTCF, WAPL, and PDS5 proteins. EMBO J..

[89] Uhlmann F., Wernic D., Poupart M.A., Koonin E.V., Nasmyth K. (2000). Cleavage of cohesin by the CD clan protease separin triggers anaphase in yeast. Cell.

[90] Uhlmann F., Lottspeich F., Nasmyth K. (1999). Sister-chromatid separation at anaphase onset is promoted by cleavage of the cohesin subunit Scc1. Nature.

[91] Waizenegger I.C., Hauf S., Meinke A., Peters J.M. (2000). Two distinct pathways remove mammalian cohesin from chromosome arms in prophase and from centromeres in anaphase. Cell.

[92] Canudas S., Smith S. (2009). Differential regulation of telomere and centromere cohesion by the Scc3 homologues SA1 and SA2, respectively, in human cells. J. Cell Biol.

[93] Carretero M., Ruiz-Torres M., Rodriguez-Corsino M., Barthelemy I., Losada A. (2013). Pds5B is required for cohesion establishment and Aurora B accumulation at centromeres. EMBO J..

[94] Dreier M.R., Bekier M.E., Taylor W.R. (2011). Regulation of sororin by Cdk1-mediated phosphorylation. J. Cell Sci..

[95] Zhang N., Panigrahi A.K., Mao Q., Pati D. (2011). Interaction of Sororin with polo-like kinase 1 mediates the resolution of chromosomal arm cohesion. J. Biol. Chem..

[96] Al-Jomah N., Mukololo L., Anjum A., Al M.M., Patel R. (2020). Pds5A and Pds5B Display Non-redundant Functions in Mitosis and Their Loss Triggers Chk1 Activation. Front Cell Dev. Biol..

[97] Rothkamm K., Kruger I., Thompson L.H., Lobrich M. (2003). Pathways of DNA double-strand break repair during the mammalian cell cycle. Mol. Cell Biol..

[98] Kowalczykowski S.C. (2015). An Overview of the Molecular Mechanisms of Recombinational DNA Repair. Cold Spring Harb. Perspect. Biol..

[99] Birkenbihl R.P., Subramani S. (1992). Cloning and characterization of rad21 an essential gene of Schizosaccharomyces pombe involved in DNA double-strand-break repair. Nucleic Acids Res..

[100] Sjogren C., Nasmyth K. (2001). Sister chromatid cohesion is required for postreplicative double-strand break repair in Saccharomyces cerevisiae. Curr. Biol..

[101] Atienza J.M., Roth R.B., Rosette C., Smylie K.J., Kammerer S., Rehbock J., Ekblom J., Denissenko M.F. (2005). Suppression of RAD21 gene expression decreases cell growth and enhances cytotoxicity of etoposide and bleomycin in human breast cancer cells. Mol. Cancer Ther..

[102] Sonoda E., Matsusaka T., Morrison C., Vagnarelli P., Hoshi O., Ushiki T., Nojima K., Fukagawa T., Waizenegger I.C., Peters J.M. (2001). Scc1/Rad21/Mcd1 is required for sister chromatid cohesion and kinetochore function in vertebrate cells. Dev. Cell.

[103] Schar P., Fasi M., Jessberger R. (2004). SMC1 coordinates DNA double-strand break repair pathways. Nucleic Acids Res..

[104] Kitagawa R., Bakkenist C.J., McKinnon P.J., Kastan M.B. (2004). Phosphorylation of SMC1 is a critical downstream event in the ATM-NBS1-BRCA1 pathway. Genes Dev..

[105] Kim S.T., Xu B., Kastan M.B. (2002). Involvement of the cohesin protein, Smc1, in Atm-dependent and independent responses to DNA damage. Genes Dev..

[106] Luo H., Li Y., Mu J.J., Zhang J., Tonaka T., Hamamori Y., Jung S.Y., Wang Y., Qin J. (2008). Regulation of intra-S phase checkpoint by ionizing radiation (IR)-dependent and IR-independent phosphorylation of SMC3. J. Biol. Chem..

[107] Yazdi P.T., Wang Y., Zhao S., Patel N., Lee E.Y., Qin J. (2002). SMC1 is a downstream effector in the ATM/NBS1 branch of the human S-phase checkpoint. Genes Dev..

[108] Watrin E., Peters J.M. (2009). The cohesin complex is required for the DNA damage-induced G2/M checkpoint in mammalian cells. EMBO J..

[109] Meisenberg C., Pinder S.I., Hopkins S.R., Wooller S.K., Benstead-Hume G., Pearl F.M.G., Jeggo P.A., Downs J.A. (2019). Repression of Transcription at DNA Breaks Requires Cohesin throughout Interphase and Prevents Genome Instability. Mol. Cell.

[110] Strom L., Lindroos H.B., Shirahige K., Sjogren C. (2004). Postreplicative recruitment of cohesin to double-strand breaks is required for DNA repair. Mol. Cell.

[111] Strom L., Karlsson C., Lindroos H.B., Wedahl S., Katou Y., Shirahige K., Sjogren C. (2007). Postreplicative formation of cohesion is required for repair and induced by a single DNA break. Science.

[112] Unal E., rbel-Eden A., Sattler U., Shroff R., Lichten M., Haber J.E., Koshland D. (2004). DNA damage response pathway uses histone modification to assemble a double-strand break-specific cohesin domain. Mol. Cell.

[113] Unal E., Heidinger-Pauli J.M., Koshland D. (2007). DNA double-strand breaks trigger genome-wide sister-chromatid cohesion through Eco1 (Ctf7). Science.

[114] Lightfoot J., Testori S., Barroso C., Martinez-Perez E. (2011). Loading of meiotic cohesin by SCC-2 is required for early processing of DSBs and for the DNA damage checkpoint. Curr. Biol..

[115] Wang S.W., Read R.L., Norbury C.J. (2002). Fission yeast Pds5 is required for accurate chromosome segregation and for survival after DNA damage or metaphase arrest. J. Cell Sci..

[116] Jin H., Guacci V., Yu H.G. (2009). Pds5 is required for homologue pairing and inhibits synapsis of sister chromatids during yeast meiosis. J. Cell Biol..

[117] Pradillo M., Knoll A., Oliver C., Varas J., Corredor E., Puchta H., Santos J.L. (2015). Involvement of the Cohesin Cofactor PDS5 (SPO76) During Meiosis and DNA Repair in Arabidopsis thaliana. Front Plant Sci..

[118] Kusch T. (2015). Brca2-Pds5 complexes mobilize persistent meiotic recombination sites to the nuclear envelope. J. Cell Sci..

[119] Buisson R., Dion-Cote A.M., Coulombe Y., Launay H., Cai H., Stasiak A.Z., Stasiak A., Xia B., Masson J.Y. (2010). Cooperation of breast cancer proteins PALB2 and piccolo BRCA2 in stimulating homologous recombination. Nat. Struct. Mol. Biol..

[120] Buisson R., Masson J.Y. (2012). PALB2 self-interaction controls homologous recombination. Nucleic Acids Res..

[121] Thomas-Claudepierre A.S., Schiavo E., Heyer V., Fournier M., Page A., Robert I., Reina-San-Martin B. (2013). The cohesin complex regulates immunoglobulin class switch recombination. J. Exp. Med..

[122] Ball A.R., Chen Y.Y., Yokomori K. (2014). Mechanisms of cohesin-mediated gene regulation and lessons learned from cohesinopathies. Biochim. Biophys. Acta.

[123] Dauban L., Montagne R., Thierry A., Lazar-Stefanita L., Bastie N., Gadal O., Cournac A., Koszul R., Beckouet F. (2020). Regulation of Cohesin-Mediated Chromosome Folding by Eco1 and Other Partners. Mol. Cell.

[124] Dorsett D., Eissenberg J.C., Misulovin Z., Martens A., Redding B., McKim K. (2005). Effects of sister chromatid cohesion proteins on cut gene expression during wing development in Drosophila. Development.

[125] Kanke M., Tahara E., Huis In’t Veld P.J., Nishiyama T. (2016). Cohesin acetylation and Wapl-Pds5 oppositely regulate translocation of cohesin along DNA. EMBO J..

[126] Nagano T., Lubling Y., Varnai C., Dudley C., Leung W., Baran Y., Mendelson C.N., Wingett S., Fraser P., Tanay A. (2017). Cell-cycle dynamics of chromosomal organization at single-cell resolution. Nature.

[127] Lieberman-Aiden E., van Berkum N.L., Williams L., Imakaev M., Ragoczy T., Telling A., Amit I., Lajoie B.R., Sabo P.J., Dorschner M.O. (2009). Comprehensive mapping of long-range interactions reveals folding principles of the human genome. Science.

[128] Rao S.S., Huntley M.H., Durand N.C., Stamenova E.K., Bochkov I.D., Robinson J.T., Sanborn A.L., Machol I., Omer A.D., Lander E.S. (2014). A 3D map of the human genome at kilobase resolution reveals principles of chromatin looping. Cell.

[129] Sanborn A.L., Rao S.S., Huang S.C., Durand N.C., Huntley M.H., Jewett A.I., Bochkov I.D., Chinnappan D., Cutkosky A., Li J. (2015). Chromatin extrusion explains key features of loop and domain formation in wild-type and engineered genomes. Proc. Natl. Acad. Sci. USA.

[130] Narendra V., Rocha P.P., An D., Raviram R., Skok J.A., Mazzoni E.O., Reinberg D. (2015). CTCF establishes discrete functional chromatin domains at the Hox clusters during differentiation. Science.

[131] Nora E.P., Lajoie B.R., Schulz E.G., Giorgetti L., Okamoto I., Servant N., Piolot T., van Berkum N.L., Meisig J., Sedat J. (2012). Spatial partitioning of the regulatory landscape of the X-inactivation centre. Nature.

[132] Merkenschlager M., Nora E.P. (2016). CTCF and Cohesin in Genome Folding and Transcriptional Gene Regulation. Annu. Rev. Genomics Hum. Genet..

[133] Nora E.P., Goloborodko A., Valton A.L., Gibcus J.H., Uebersohn A., Abdennur N., Dekker J., Mirny L.A., Bruneau B.G. (2017). Targeted Degradation of CTCF Decouples Local Insulation of Chromosome Domains from Genomic Compartmentalization. Cell.

[134] Anderson E.C., Nora E.P. (2020). Setting new boundaries with transcription and CTCF. Nat. Genet..

[135] Nora E.P., Caccianini L., Fudenberg G., So K., Kameswaran V., Nagle A., Uebersohn A., Hajj B., Saux A.L., Coulon A. (2020). Molecular basis of CTCF binding polarity in genome folding. Nat. Commun..

[136] de Wit E., Vos E.S., Holwerda S.J., Valdes-Quezada C., Verstegen M.J., Teunissen H., Splinter E., Wijchers P.J., Krijger P.H., de L.W. (2015). CTCF Binding Polarity Determines Chromatin Looping. Mol. Cell.

[137] Wendt K.S., Peters J.M. (2009). How cohesin and CTCF cooperate in regulating gene expression. Chromosome. Res..

[138] Dorsett D., Merkenschlager M. (2013). Cohesin at active genes: A unifying theme for cohesin and gene expression from model organisms to humans. Curr. Opin. Cell Biol..

[139] Kagey M.H., Newman J.J., Bilodeau S., Zhan Y., Orlando D.A., van Berkum N.L., Ebmeier C.C., Goossens J., Rahl P.B., Levine S.S. (2010). Mediator and cohesin connect gene expression and chromatin architecture. Nature.

[140] Parelho V., Hadjur S., Spivakov M., Leleu M., Sauer S., Gregson H.C., Jarmuz A., Canzonetta C., Webster Z., Nesterova T. (2008). Cohesins functionally associate with CTCF on mammalian chromosome arms. Cell.

[141] Wendt K.S., Yoshida K., Itoh T., Bando M., Koch B., Schirghuber E., Tsutsumi S., Nagae G., Ishihara K., Mishiro T. (2008). Cohesin mediates transcriptional insulation by CCCTC-binding factor. Nature.

[142] Rao S.S.P., Huang S.C., Glenn St H.B., Engreitz J.M., Perez E.M., Kieffer-Kwon K.R., Sanborn A.L., Johnstone S.E., Bascom G.D., Bochkov I.D. (2017). Cohesin Loss Eliminates All Loop Domains. Cell.

[143] Fudenberg G., Imakaev M., Lu C., Goloborodko A., Abdennur N., Mirny L.A. (2016). Formation of Chromosomal Domains by Loop Extrusion. Cell Rep..

[144] Vietri R.M., Barrington C., Henderson S., Ernst C., Odom D.T., Tanay A., Hadjur S. (2015). Comparative Hi-C reveals that CTCF underlies evolution of chromosomal domain architecture. Cell Rep..

[145] Kubo N., Ishii H., Xiong X., Bianco S., Meitinger F., Hu R., Hocker J.D., Conte M., Gorkin D., Yu M. (2021). Promoter-proximal CTCF binding promotes distal enhancer-dependent gene activation. Nat. Struct. Mol. Biol..

[146] Gandhi R., Gillespie P.J., Hirano T. (2006). Human Wapl is a cohesin-binding protein that promotes sister-chromatid resolution in mitotic prophase. Curr. Biol..

[147] Kueng S., Hegemann B., Peters B.H., Lipp J.J., Schleiffer A., Mechtler K., Peters J.M. (2006). Wapl controls the dynamic association of cohesin with chromatin. Cell.

[148] Misulovin Z., Pherson M., Gause M., Dorsett D. (2018). Brca2, Pds5 and Wapl differentially control cohesin chromosome association and function. PLoS Genet..

[149] Wutz G., Ladurner R., St Hilaire B.G., Stocsits R.R., Nagasaka K., Pignard B., Sanborn A., Tang W., Varnai C., Ivanov M.P. (2020). ESCO1 and CTCF enable formation of long chromatin loops by protecting cohesin(STAG1) from WAPL. Elife..

[150] Carvajal-Maldonado D., Byrum A.K., Jackson J., Wessel S., Lemacon D., Guitton-Sert L., Quinet A., Tirman S., Graziano S., Masson J.Y. (2019). Perturbing cohesin dynamics drives MRE11 nuclease-dependent replication fork slowing. Nucleic Acids Res..

[151] Barbero J.L. (2013). Genetic basis of cohesinopathies. Appl. Clin. Genet..

[152] Deardorff M.A., Kaur M., Yaeger D., Rampuria A., Korolev S., Pie J., Gil-Rodriguez C., Arnedo M., Loeys B., Kline A.D. (2007). Mutations in cohesin complex members SMC3 and SMC1A cause a mild variant of cornelia de Lange syndrome with predominant mental retardation. Am. J. Hum. Genet..

[153] Krantz I.D., McCallum J., Descipio C., Kaur M., Gillis L.A., Yaeger D., Jukofsky L., Wasserman N., Bottani A., Morris C.A. (2004). Cornelia de Lange syndrome is caused by mutations in NIPBL, the human homolog of Drosophila melanogaster Nipped-B. Nat. Genet..

[154] Musio A., Selicorni A., Focarelli M.L., Gervasini C., Milani D., Russo S., Vezzoni P., Larizza L. (2006). X-linked Cornelia de Lange syndrome owing to SMC1L1 mutations. Nat. Genet..

[155] Tonkin E.T., Wang T.J., Lisgo S., Bamshad M.J., Strachan T. (2004). NIPBL, encoding a homolog of fungal Scc2-type sister chromatid cohesion proteins and fly Nipped-B, is mutated in Cornelia de Lange syndrome. Nat. Genet..

[156] Kline A.D., Moss J.F., Selicorni A., Bisgaard A.M., Deardorff M.A., Gillett P.M., Ishman S.L., Kerr L.M., Levin A.V., Mulder P.A. (2018). Diagnosis and management of Cornelia de Lange syndrome: First international consensus statement. Nat. Rev. Genet..

[157] Jackson L., Kline A.D., Barr M.A., Koch S. (1993). de Lange syndrome: A clinical review of 310 individuals. Am. J. Med. Genet..

[158] Gillis L.A., McCallum J., Kaur M., Descipio C., Yaeger D., Mariani A., Kline A.D., Li H.H., Devoto M., Jackson L.G. (2004). NIPBL mutational analysis in 120 individuals with Cornelia de Lange syndrome and evaluation of genotype-phenotype correlations. Am. J. Hum. Genet..

[159] Murthy S., Agoulnik I.U., Weigel N.L. (2005). Androgen receptor signaling and vitamin D receptor action in prostate cancer cells. Prostate.

[160] Harada H., Uchida N., Shimada Y., Kumimoto H., Shinoda M., Imamura M., Ishizaki K. (2001). Polymorphism and allelic loss at the AS3 locus on 13q12-13 in esophageal squamous cell carcinoma. Int. J. Oncol..

[161] Geck P., Maffini M.V., Szelei J., Sonnenschein C., Soto A.M. (2000). Androgen-induced proliferative quiescence in prostate cancer cells: The role of AS3 as its mediator. Proc. Natl. Acad. Sci. USA.

[162] Geck P., Sonnenschein C., Soto A.M. (2001). The D13S171 marker, misannotated to BRCA2, links the AS3 gene to various cancers. Am. J. Hum. Genet..

[163] Maffini M.V., Geck P., Powell C.E., Sonnenschein C., Soto A.M. (2002). Mechanism of androgen action on cell proliferation: AS3 protein as a mediator of proliferative arrest in the rat prostate. Endocrinology.

[164] Kim M.S., An C.H., Yoo N.J., Lee S.H. (2013). Frameshift mutations of chromosome cohesion-related genes SGOL1 and PDS5B in gastric and colorectal cancers with high microsatellite instability. Hum. Pathol..

[165] Denes V., Pilichowska M., Makarovskiy A., Carpinito G., Geck P. (2010). Loss of a cohesin-linked suppressor APRIN (Pds5b) disrupts stem cell programs in embryonal carcinoma: An emerging cohesin role in tumor suppression. Oncogene.

[166] Brough R., Bajrami I., Vatcheva R., Natrajan R., Reis-Filho J.S., Lord C.J., Ashworth A. (2012). APRIN is a cell cycle specific BRCA2-interacting protein required for genome integrity and a predictor of outcome after chemotherapy in breast cancer. EMBO J..

[167] Ma J., Cui Y., Cao T., Xu H., Shi Y., Xia J., Tao Y., Wang Z.P. (2019). PDS5B regulates cell proliferation and motility via upregulation of Ptch2 in pancreatic cancer cells. Cancer Lett..

[168] Sohn M.S., Kang M., Kang S.M., Bae S. (2021). Downregulation of APRIN expression increases cancer cell proliferation via an interleukin-6/STAT3/cyclin D axis. Oncol. Lett..

[169] Debernardi S., Massat N.J., Radon T.P., Sangaralingam A., Banissi A., Ennis D.P., Dowe T., Chelala C., Pereira S.P., Kocher H.M. (2015). Noninvasive urinary miRNA biomarkers for early detection of pancreatic adenocarcinoma. Am. J. Cancer Res..

[170] Wang L., Zheng J., Sun C., Wang L., Jin G., Xin L., Jin Z., Wang D., Li Z. (2017). MicroRNA expression levels as diagnostic biomarkers for intraductal papillary mucinous neoplasm. Oncotarget..

[171] Komatsu S., Ichikawa D., Miyamae M., Kawaguchi T., Morimura R., Hirajima S., Okajima W., Ohashi T., Imamura T., Konishi H. (2015). Malignant potential in pancreatic neoplasm; new insights provided by circulating miR-223 in plasma. Expert. Opin. Biol. Ther..

[172] Tachibana H., Sho R., Takeda Y., Zhang X., Yoshida Y., Narimatsu H., Otani K., Ishikawa S., Fukao A., Asao H. (2016). Circulating miR-223 in Oral Cancer: Its Potential as a Novel Diagnostic Biomarker and Therapeutic Target. PLoS ONE.

[173] Fassan M., Saraggi D., Balsamo L., Realdon S., Scarpa M., Castoro C., Coati I., Salmaso R., Farinati F., Guzzardo V. (2017). Early miR-223 Upregulation in Gastroesophageal Carcinogenesis. Am. J. Clin. Pathol..

[174] Bagheri A., Khorram Khorshid H.R., Mowla S.J., Mohebbi H.A., Mohammadian A., Yaseri M., Solaymani-Dodaran M., Sherafatian M., Tavallaie M. (2017). Altered miR-223 Expression in Sputum for Diagnosis of Non-Small Cell Lung Cancer. Avicenna. J. Med. Biotechnol..

[175] Pomari E., Lovisa F., Carraro E., Primerano S., D’Amore E.S.G., Bonvini P., Nigro L.L., Vito R., Vinti L., Farruggia P. (2017). Clinical impact of miR-223 expression in pediatric T-Cell lymphoblastic lymphoma. Oncotarget..

[176] Schultz N.A., Dehlendorff C., Jensen B.V., Bjerregaard J.K., Nielsen K.R., Bojesen S.E., Calatayud D., Nielsen S.E., Yilmaz M., Hollander N.H. (2014). MicroRNA biomarkers in whole blood for detection of pancreatic cancer. JAMA.

[177] He D., Huang C., Zhou Q., Liu D., Xiong L., Xiang H., Ma G., Zhang Z. (2017). HnRNPK/miR-223/FBXW7 feedback cascade promotes pancreatic cancer cell growth and invasion. Oncotarget..

[178] Ma J., Cao T., Cui Y., Zhang F., Shi Y., Xia J., Wang Z.P. (2019). miR-223 Regulates Cell Proliferation and Invasion via Targeting PDS5B in Pancreatic Cancer Cells. Mol. Ther. Nucleic Acids.

[179] Kumar D., Sakabe I., Patel S., Zhang Y., Ahmad I., Gehan E.A., Whiteside T.L., Kasid U. (2004). SCC-112, a novel cell cycle-regulated molecule, exhibits reduced expression in human renal carcinomas. Gene.

[180] Hagemann C., Weigelin B., Schommer S., Schulze M., Al-Jomah N., Anacker J., Gerngras S., Kuhnel S., Kessler A.F., Polat B. (2011). The cohesin-interacting protein, precocious dissociation of sisters 5A/sister chromatid cohesion protein 112, is up-regulated in human astrocytic tumors. Int. J. Mol. Med..

[181] Zheng M.Z., Zheng L.M., Zeng Y.X. (2008). SCC-112 gene is involved in tumor progression and promotes the cell proliferation in G2/M phase. J. Cancer Res. Clin. Oncol..

[182] Viera A., Berenguer I., Ruiz-Torres M., Gomez R., Guajardo A., Barbero J.L., Losada A., Suja J.A. (2020). PDS5 proteins regulate the length of axial elements and telomere integrity during male mouse meiosis. EMBO Rep..

[183] Liu Y., Xu H., Van der Jeught K., Li Y., Liu S., Zhang L., Fang Y., Zhang X., Radovich M., Schneider B.P. (2018). Somatic mutation of the cohesin complex subunit confers therapeutic vulnerabilities in cancer. J. Clin. Investig..

[184] van der Lelij P., Newman J.A., Lieb S., Jude J., Katis V., Hoffmann T., Hinterndorfer M., Bader G., Kraut N., Pearson M.A. (2020). STAG1 vulnerabilities for exploiting cohesin synthetic lethality in STAG2-deficient cancers. Life Sci. Alliance.

[185] van der Lelij P., Lieb S., Jude J., Wutz G., Santos C.P., Falkenberg K., Schlattl A., Ban J., Schwentner R., Hoffmann T. (2017). Synthetic lethality between the cohesin subunits STAG1 and STAG2 in diverse cancer contexts. Elife.

[186] Benedetti L., Cereda M., Monteverde L., Desai N., Ciccarelli F.D. (2017). Synthetic lethal interaction between the tumour suppressor STAG2 and its paralog STAG1. Oncotarget.

